# Evaluation of the Efficacy of Epiduroscopic Laser Neural Discectomy in Lumbar Disc Herniations: Retrospective Analysis of 163 Cases- Evaluation of the Efficacy of ELNP

**DOI:** 10.1155/2020/7361691

**Published:** 2020-10-29

**Authors:** Ali Metin Ülgen, Serbülent Gökhan Beyaz, Mustafa Erkan Inanmaz, Fatih Şahin

**Affiliations:** ^1^Department of Anesthesiology and Pain Medicine, Sakarya University Training and Research Hospital, Serdivan, Sakarya, Turkey; ^2^Department of Anesthesiology and Pain Medicine, Sakarya University Faculty of Medicine, Serdivan, Sakarya, Turkey; ^3^Department of Orthopedics and Traumatology, Sakarya University Faculty of Medicine, Serdivan, Sakarya, Turkey; ^4^Department of Anesthesiology and Pain Medicine, Sakarya University Training and Research Hospital, Serdivan, Sakarya, Turkey

## Abstract

**Background:**

Epiduroscopy, or spinal endoscopy, is the visualisation of the epidural space using a percutaneous and minimally invasive imaging fiberoptic device. Recently, as a result of some studies, it has been reported that laser therapy with epiduroscopic laser neural discectomy (ELND) was applied during multiple lesions.

**Methods:**

In this study, ELND performed between January 2012 and July 2016 at the Algology Clinic of the Department of Anesthesiology and Reanimation, Sakarya University Training and Research Hospital, was examined retrospectively. The Oswestry Disability Index (ODI) and Visual Analogue Scale (VAS) scores were recorded preoperatively, as well as after 2 weeks and 2, 6, and 12 months after the ELND.

**Results:**

According to the preoperative VAS and ODI scores, the decrease in postoperative 2nd week, 2nd, 6th, and 12th month VAS and ODI scores was significant (*p*=0.001). Similarly, according to the postoperative 2nd week VAS and ODI scores, decrease in postoperative 6th and 12th VAS and ODI scores was significant (*p*=0.001).

**Conclusions:**

As a result, ELND with Holmium: YAG laser, which is a new technique in patients with lumbar disc herniated low back and/or leg pain, can reduce VAS and ODI scores from 2 weeks without any complications that open surgery can bring with it. We believe that it is a useful and advanced technique in treatment of lumbar disc herniation and has low complication rates that provides maximum efficacy from the first year.

## 1. Introduction

Epiduroscopy, or spinal endoscopy, is the visualisation of the epidural space using a percutaneous and minimally invasive imaging fiberoptic device. The procedure involves the placement of a tiny fiberoptic camera, with the help of a catheter, in the epidural space via a small incision in the sacral hiatus. Relevant areas can be imaged, adhesions can be opened, and steroids can be injected with a local anesthetic [1, 2]. Epiduroscopy is a therapeutic method for the new diagnosis of low back pain, especially radicular pain [3].

Epiduroscopy is increasingly important not only in definitive diagnosis but also in treatment of chronic low back and leg pain. Recently, it has been reported that laser therapy with epiduroscopic laser neural discectomy (ELND) is applied during multiple lesions [4]. With ELND, pathologies such as herniated disc and adhesions can be treated directly. The volume of the herniated disc is reduced and adhesions are removed by laser [5]. When clinical and/or radiological findings conflict, epiduroscopy should be used as early as possible to counteract the chronicity of pain in patients with spinal pain syndrome [1, 2]. We aimed to retrospectively review the efficacy of ELND performed in our clinic.

## 2. Material and Methods

In this study, ELND performed between January 2012 and July 2016 at the Algology Clinic of the Department of Anesthesiology and Reanimation, Sakarya University Training and Research Hospital, was examined retrospectively. After approval was received from the Sakarya University Faculty of Medicine's Ethics Committee, patient information was obtained from the KarMed (Kardelen Software, Turkey) hospital information system. Files were scanned from the hospital archive. The following factors were examined: the age, gender, weight, lumbar disc herniation level, preoperative and postoperative surgical history, amount of physiological saline used, duration of the procedure, lumbar epidural steroid (LES) usage, epiduroscopic laser power, and complications after the procedure. The Oswestry Disability Index (ODI) and Visual Analogue Scale (VAS) scores were recorded preoperatively, as well as after 2 weeks and 2, 6, and 12 months postoperatively from the patient files. Inclusion criteria were patients who had experienced low back/leg pain for at least 3 months, had not benefitted from conservative treatment, had no motor deficit in the examination, had not accepted open surgery, and had an MSU classification 2A, 2B, 2C, and 2AB herniated disc in the axial section as confirmed by MRI. Exclusion criteria were patients who had extraforaminal disc extrudation in the axial section as confirmed by MRI, had previously undergone endoscopic and/or open lumbar surgery, had a bleeding diathesis as confirmed by preoperative laboratory tests, had a local infection at the site of intervention, or had a systemic infection.

Preoperatively, the 18 G vascular access was opened; 1 g of ceftriaxone (Rocephin) was administered 1 hour prior to the procedure, and the patient was taken to the operating table. Following this, a 0.9% saline infusion was started intravenously. The patient was monitored by standardized monitoring techniques, which are electrocardiography, noninvasive blood pressure, and pulse oximetry.

Aseptic conditions were maintained after cleaning the interference area. After the patient was covered with a sterile cover, caudal region anesthesia was provided with a 22 G Quincke spinal needle. Intervention was performed with the trocar from the sacral hiatus. The epidurography, carried out with C-arm scopy, revealed that the trocar was in the midline. The Spinaut-V ® video guided catheter (IMEDICOM Co.Ltd, Gyeonggi-do, Republic of Korea) was directed to the anterior epidural space, where the lumbar disc lesion was present. Saline infusion to the epidural space was performed at the rate of 0.15–0.20 mL/sec during the procedure. After the herniated disc was visualized, a laser probe was placed in the disc. Following this, the Holmium laser was applied, and the data that emerged during the decompression were recorded. If there was disc herniation in another region, the video-guided catheter was advanced to the target site with the help of epiduroscopic imaging. If there was no other hernia, the procedure was terminated after a lumbar epidural steroid injection (16 mg of dexamethasone with 15 mg of bupivacaine in a 7–10 mL volume). Finally, epidurography was performed to evaluate the effectiveness of the procedure.

Pain and disability assessments were performed based on Visual Analogue Scale (VAS) and the Oswestry Disability Index (ODI). The selected patients were evaluated before the procedure, at the 2nd week after the procedure, and at the 2nd, 6th, and 12th months, and the pain and disability scales were applied by policlinic controls and by telephone.

In this study,

1. compared to the preoperative VAS scores, postoperative 12th month VAS scores decreased by 50%
2. the postoperative 12th month VAS scores fell to 3 and below
3. compared to the preoperative ODI scores, a decrease of 40% or more was observed in postoperative 12th month ODI scores
4. the postoperative 12th month ODI scores were below 40% and were evaluated as successful

The patient progress levels in the hospital system and files were scanned. Complications (bleeding, infection, dural puncture, and neurological signs) that emerged during and after the procedure were recorded.

## 3. Statistical Analysis

The 2007 Number Cruncher Statistical System (NCSS, Kaysville, Utah, USA) program was used for statistical analysis. The Student *t*-test and Mann–Whitney *U*-test were used for descriptive statistical methods (mean, standard deviation, median, frequency, ratio, minimum, and maximum) in two groups, as well as for the normal distribution of quantitative data. The Kruskal–Wallis test and Mann–Whitney *U*-test were used to compare the three groups with no normal distribution. The Pearson Chi-square test was used to evaluate qualitative data, and Spearman's correlation analysis was used to evaluate intervariable relationships. The Friedman and Wilcoxon signed ranks tests were used in the evaluation of three or more follow-up parameters without normal distribution. Diagnostic screening tests (sensitivity and specificity) and receiver operating characteristic (ROC) analysis were used to determine the cutoff value for age. Significance was evaluated at the level of (*p* < 0.01).

## 4. Results

Data for 190 patients were attained as a result of scanning the hospital system. Four patients were excluded from the study because they did not want to participate, nine patients could not be treated because of anatomical difficulty (failure of the trocar to pass the sacral hiatus), and 14 patients could not be reached via telephone or other communication methods. Thus, 163 patients were included in the study. The distribution of the demographic characteristics of the patients is shown in Table 1. Preoperative characteristics and complications of epiduroscopic discectomy procedures are also shown in Table 2.

**Figure 1:**
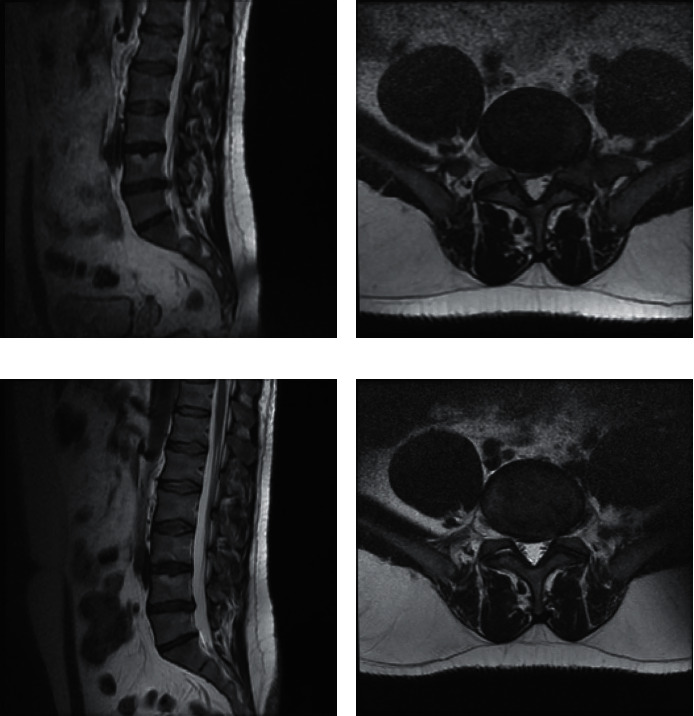
Preoperatively and postoperatively axial and sagittal images of MRI before/after Holmium-YAG laser-assisted ELND. (a) Preoperative sagittal MRI image of the patient. (b) Preoperative axial MRI image of the patient. (c) Postoperative sagittal MRI image of the patient. (d) Postoperative axial MRI image of the patient.

When lumbar MRI images of the patients were examined, protrusion was observed (100%, *n* = 163, Figure 1). The percentages for different hernia types were 15.3% (*n* = 25) for L3-L4, 75.5% (*n* = 123) for L4-L5, and 63.8% (*n* = 104) for L5-S1. The percentages for patients with more than one level of hernia were 33.1 % (*n* = 53).

**Figure 2:**
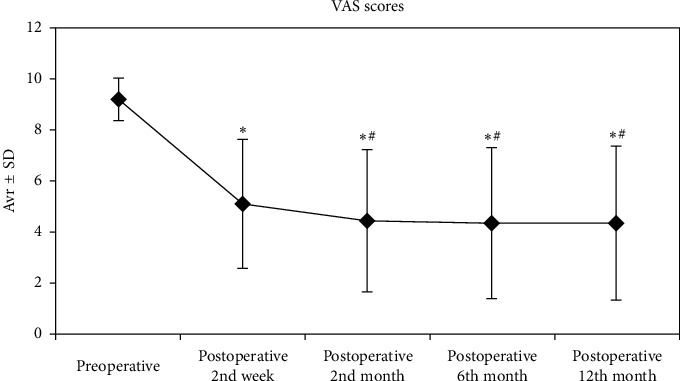
VAS values were taken during 12 months preoperatively and postoperatively. ^*∗*^: statistically significant compared to preoperative values (*p* < 0,01). ^#^: statistically significant compared to postoperative 2nd week (*p* < 0.01). Friedman test and Wilcoxon signed ranks test, *p* < 0.01.

The VAS scores of the patients were evaluated, and changes in the preoperative scores and the postoperative 2nd week and 2nd, 6th, and 12th month scores were statistically significant (*p*=0.001). Compared to the preoperative VAS scores, the decreases in postoperative 2nd week and 2nd, 6th, and 12th month VAS scores were significant (*p*=0.001). Similarly, compared to the postoperative 2nd week VAS scores, the decreases in postoperative 6th and 12th VAS scores were significant (*p*=0.001). No significant decreases (respectively, *p*=0.309, *p*=0.176, and *p*=0.180; see Figure 2) were observed comparing postoperative 6th month and 12th month VAS scores to postoperative 2nd month scores.

**Figure 3:**
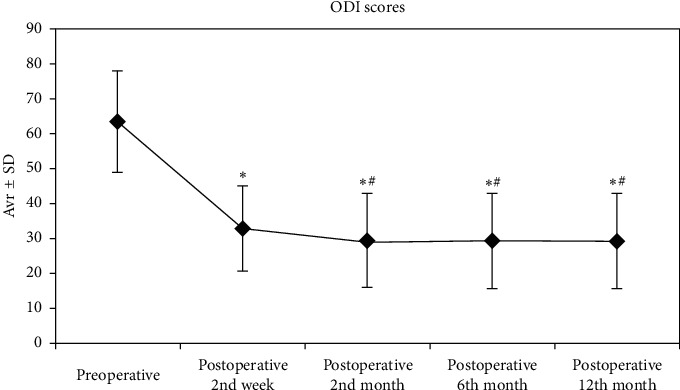
. ODI values were taken during 12 months preoperatively and postoperatively. ^*∗*^: statistically significant compared to preoperative values (*p* < 0,01). ^#^: statistically significant compared to postoperative 2nd week (*p* < 0.01). Friedman test and Wilcoxon signed ranks test, *p* < 0.01.

The ODI scores of the patients were evaluated, and the changes in the preoperative ODI scores and the postoperative 2nd week and 2nd, 6th, and 12th month ODI scores were statistically significant (*p*=0.001). Compared to the preoperative ODI scores, the decreases in postoperative 2nd week and 2nd, 6th, and 12th month ODI scores were significant (*p*=0.001). Similarly, compared to the postoperative 2nd week ODI scores, the decreases in postoperative 6th and 12th month ODI scores were significant (*p*=0.001). Comparing the postoperative 6th and 12th month ODI scores with the postoperative 2nd month scores, no significant decreases (respectively, *p*=0.853, *p*=0.388, and *p*=0.141; Figure 3) were observed.

The postoperative 12th month VAS scores of the patients decreased by 51.72% compared to the preoperative VAS scores. Compared to the preoperative VAS scores, 63.8% (*n* = 104) of the patients postoperative 12th month VAS scores decreased by 50% or more, and 50.9% (*n* = 83) of the patients had postoperative 12th VAS scores of 3 and below.

The postoperative 12th month ODI scores of the patients decreased by 50.47% compared to the preoperative scores. Compared to the preoperative ODI scores, 73.6.8% (*n* = 120) of the patients postoperative 12th month ODI scores decreased by 40% or more, and 79.14% (*n* = 129) of the patients 12th months ODI scores were below 40%.

When the reduction in the preoperative and 2nd week and 6th and 12th month postoperative VAS and ODI scores were examined with those with those patients have single-level hernias and those with two or more levels, no significant difference was observed when comparing hernia levels (*p*=0.605 for VAS, *p*=0.244).

Ten patients had a history of preoperative lumbar laminectomy surgical history. These patients did not need surgery after the ELND. Among the 163 patients, 13 (7.9%) patients who had no pain relief and increased herniation grade were directed to surgical treatment. No significant decrease in postoperative 12th month VAS scores were seen compared with the preoperative VAS scores of the patients evaluated according to preoperative surgical history (*p*=0.704). No significant decrease in postoperative 12th month ODI scores were observed compared with the preoperative ODI scores of the patients evaluated according to preoperative surgical history (*p*=0.961).

According to the success criteria obtained from the VAS scores, a significant difference was found between the mean age of the patients (*p*=0.001). It was observed that the age average of those who showed a decrease of 50% or more in the VAS scores was lower than the average of those who showed a decrease of 50% or less. In this regard, the cutoff point for age was determined to be 44 according to VAS success (Table 3). According to the success criteria obtained from the ODI scores, a significant difference was noted among the mean ages of the patients (*p*=0.001). It was observed that the age average of those who showed a decrease of 40% or more in the ODI scores was lower than the average of those who showed a decrease of 40% or less. In this regard, the cutoff point for age was determined to be 44 according to ODI success (Table 3).

**Figure 4:**
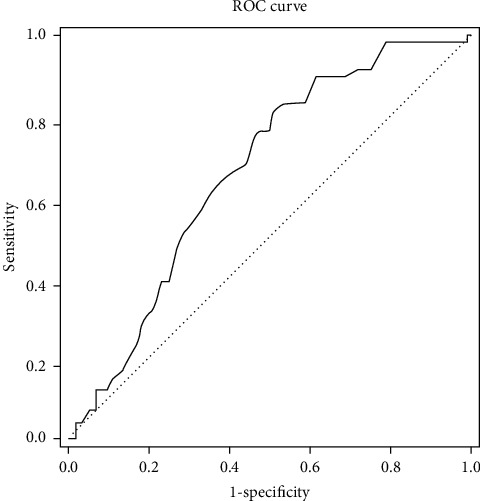
ROC analysis chart for age according to VAS success. For age 44 cutting value, sensitivity 81.36%, specificity 49.04%, the positive predictive value is 47.52, and the negative predictive value is 82.26.

**Figure 5:**
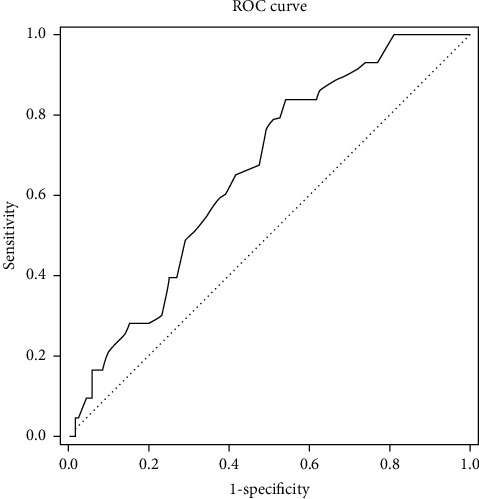
ROC analysis chart for age according to ODI success. For age 44 cutting value, sensitivity was 83.72%, specificity 45.83%, the positive predictive value is 35.64, and the negative predictive value is 88.71.

A statistically significant correlation between age and the VAS success level (*p*=0.001) was observed. The risk of under 50% of the reductions in VAS scores in patients aged 44 and older was 4.199-fold (Figure 4, Table 4). A statistically significant correlation was found between age and the ODI success level (*p*=0.001). The risk of under 40% of the reductions in ODI scores in patients 44 years old and older was 4.352-fold (Figure 5, Table 4).

## 5. Discussion

Epiduroscopy is an effective diagnostic and therapeutic method that is becoming increasingly important in patients with chronic low back and/or leg pain. Several articles that have been published in the literature show that the epiduroscopy procedure has positive effects on pain scores and quality of life [6–9]. We performed a retrospective analysis of the patients who underwent ELND with a single indication, and then, we evaluated the efficacy of the procedure.

Only four studies in the literature have reported on ELND [6–9]. Ruetten et al. [10, 11] conducted two separate prospective studies with 34 and 68 patients who did not respond to conservative treatment and who underwent ELND using Holmium: YAG laser, showing positive results of 44% and 48.5%, respectively (follow-ups of 8 weeks and 2 points of VAS reduction). Rutten et al. [10, 11] had some metadological deficiencies in their studies. Only VAS was used in the study to assess the severity of pain, and the patients were followed up for only 8 weeks. In these procedures that treat multifactorial low back pain, 8-week follow-up is not sufficient. In addition, a percentage reduction in VAS scores by 2 points was assessed in their study. In our study, the patients were followed up for 12 months to assess their VAS scores and ODI scores. Compared to the preoperative values, postoperative 12th month VAS scores decreased by 50% and postoperative 12th VAS scores decreased to 3 and below as a success criterion. The results obtained at the end of the 8-week (2-month) and 12-month follow-up were much more effective than in the work of Rutten et al. [10, 11].

Jo et al. [9] retrospectively compared the satisfaction ratings of patients after ELND. In the study, 39 patients were treated and separated into two groups: those who had a lumbar surgery history (group 1) and those who did not undergo lumbar surgery (group 2). In group 1, 16 patients showed 94.1% more healing, and in group 2, 19 patients showed 86.4% more healing. No statistically significant difference was found in the percentage of satisfaction after ELND between the two groups. ELND was satisfactory for patients with chronic low back pain and/or leg pain regardless of surgery history (85% excess). Jo et al. [9] used subjective assessments of the satisfaction ratings of the patients in their study. However, no objective scoring was used. In our study, the pain and functional status of the patients were assessed with VAS and ODI scores. Regardless of the past history of surgery, 163 patients who underwent ELND were found to have a significant decrease in preoperative and 2nd week and 2nd, 6th, and 12th month postoperative VAS and ODI scores (*p* < 0.01).

In another retrospective study where ELND was performed by Jo and Yang [5], 77 patients were evaluated. The great majority of patients had FBSS (44 patients). The improvement in pain symptoms at 2 weeks and 1 month after the procedure was assessed by dividing into five grades: very good (5), good (4), no change (3), poor (2), and very poor (1). A total of 67 patients (87.0%) had pain relief after 2 weeks, and 63 patients (81.8%) had pain relief after 1 month. Jo and Yang's [5] results were based on subjective assessments of the work. Also, the duration of the monitoring was quite short.

Ruetten et al. [10, 11] reported that the amount of laser power used in the epiduroscopy ranged from 256 to 1400 joules, with an average of 1180 joules. However, the epiduroscopic laser used in our study ranged from 85 to 858 joules, with an average of 312.31 ± 157.82 joules. In our study, we used up to one-fourth of the laser power values that Ruetten et al. [10, 11] used, and more favorable results were obtained.

Donato et al. [8] evaluated the efficacy of epiduroscopy in a prospective study followed for 48 months; 234 patients with chronic low back pain who continued pain despite conservative treatment were included in the study. VAS and ODI scores of the patients were examined. In the study, the epidural space was washed with an intermittent infusion of saline solution and 150 UI hyaluronidase, followed by the application of ozone (8 mL; 38 *γ*/mL) and ciprofloxacin 50 mg. Prospective evaluations of short- and long-term efficacy (1st week and 3rd, 6th, 12th, 24th, 36th, and 48th months) were considered positive with VAS scores <5 and ODI scores <40%. The treatment significantly reduced the VAS scores from the first week and the ODI scores from the third month (*p* < 0.01). In their study, 66% of the patients had VAS scores below 5, and 78% of the patients had ODI scores below 40%. The application of ciprofloxacin and ozone to the targeted area of the epidural space and mechanical adhesiolysis has been reported to be an effective technique for providing good analgesic action to improve VAS and ODI in chronic low back pain. In our study, significant decreases were found in the VAS and ODI scores from the 2nd week (*p* < 0.01). In total, 50.9% of the patients' postoperative 12th month VAS scores had fallen to 3 and below, and 79.14% of the patients' postoperative 12th month ODI scores were found to be below 40%. Our study and Donato et al.'s work [8] were not similar in terms of the patient selection criteria. Donato et al. [8] performed injections of ciprofloxacin with an oxygen (O_2_) and ozone (O_3_) mixture for epidural lysis. In our study, treatments (e.g., O_3_ and ciprofloxacin) other than laser applications were not applied during epiduroscopy.

Helm et al. [12] determined clinically significant improvement (pain relief in low back and/or lower extremity pain) by over 50% in the VAS scores in epiduroscopy procedures performed in patients with laminectomy history. The VAS scores of the patients included in the study were found to decrease by 71% in the 1st month, 63% in the 3rd and 6th month, and 38% in the 12th month. A comparison of our retrospective study with this study is rather difficult. In our study, the epiduroscopy procedure was applied because of the epidural adhesions and new disc pathologies that occurred after open surgery in 10 patients, and no statistically significant benefit was obtained (*p* > 0.05).

Donato et al. [7] conducted a study of 350 patients with the longest follow-up (60 months) in the literature. In their study, patient selection criteria were broad, in that patients with FBSS, spondylolisthesis, stenosis, and disc herniation were included in the study. When the study's methodology was examined, postoperative VAS scores <5 and ODI scores <40% were evaluated as a good result. Postoperative VAS scores were <5 in 65% of patients, and postoperative ODI scores were <40% in 78% of patients. Although the selection criteria for patients were broad, very successful results were obtained. Laminectomy story (6.1%) was low in our study, and patients with low back and/or leg pain due to disc herniation were included in the study. The patient selection criteria must be similar to compare both successful studies, even though the results are close to each other.

Manchikanti et al. [6] included patients with chronic pain who did not respond to conservative treatment strategies, including epidural injections with C-arm scopy and percutaneous adhesiolysis, in a prospective, randomized, double-blind study. Most of the patients who were divided into two groups had a surgical history (group 1: 73%; group 2: 84%). In group 1 (the control group), the epiduroscope was brought to a sacral channel level, and a corticosteroid and local anesthetic mixture was applied. In group 2, the same corticosteroid and local anesthetic mixture was applied after epiduroscopy and appropriate adhesiolysis in the target area. In Group 2, 23 patients (13%) had significantly improved VAS and ODI scores after the 1st, 3rd, and 6th months (57%) (*p* < 0.01). All other outcome measures, including psychometric tests, were significantly improved at the end of the 1st, 3rd, and 6th months. In the control group, the same parameters improved only at the end of the first month, but no improvement was observed in the following months. Manchikanti et al. [6] reported that epiduroscopy was an effective treatment for the patients who do not adequately respond to epidural injections and percutaneous adhesions in their study. In our study, a mixture of corticosteroid and local anesthetic was administered after mechanical adhesiolysis in all patients. No corticosteroid or local anesthetic mixture was administered to 12 patients with dural puncture. The decrease in VAS and ODI scores of the patients was significant (*p* < 0.01).

In a limited number of patients with radiculopathy, a sufficient decrease in pain scores in epiduroscopy studies without laser use has been reported [13–18].

Another indication of the epiduroscopy technique is lumbar spinal stenosis. Igarashi et al. [19] studied the effect of epiduroscopy on patients with degenerative lumbar stenosis; 58 patients were separated into two groups. The monosegmental group (*n* = 34) and the multisegmental group (*n* = 24) were differentiated according to the number of affected nerve roots. When they examined the VAS scores for low back and leg pain, they found that the reduction in pain lasted 3 months in the multisegmental group and 12 months in the monosegmental group. In our study, in contrast, the patients were divided into two groups, one of which was on one level and the other on both levels. When the preoperative and 2nd week and 2nd, 6th, and 12th month postoperative VAS and ODI scores of the patients in the two groups were compared with each other, no significant difference were found in relation to the hernia levels (*p* > 0.05), indicating that the epiduroscopy technique may have another advantage.

The incidence of complications is inversely related to the practitioner's professional skills and experience. It should not be forgotten that risks are associated with medical procedures in the spinal region, even when performed properly and carefully [20]. Beyaz [20] reported on a case in which seizure activity occurred during the epiduroscopix lysis (with 110 mL of fluid in the epidural space). However, Pereira et al. [18] used up to 650 mL of saline without any complications during or after epiduroscopy. Most publications on epiduroscopy techniques have suggested that the volume of saline injected into the epidural space should be no more than 100 to 350 mL to prevent the complications associated with increased hydrostatic pressure in the epidural space [4–7, 21]. Pereira et al. [18] reported that using a large amount of physiological saline during epiduroscopy may provide additional procedural benefit. Some publications have suggested that a further theoretical benefit of using higher volumes of saline is that phospholipase-A2 in the epidural space and proinflammatory cytokines can be removed from the epidural space [15, 22]. Saline infusion was performed at the rate of 0.15–0.20 mL/sec during the epiduroscopy applications performed in our clinic. The amount of fluid given to the epidural space ranged from 40 to 280 mL, with a mean of 133.42 ± 48.88 mL. We think that these quantities are safe liquid quantities that can be applied in the epiduroscopy process.

Headaches occurred in 9 of 12 patients with dural puncture. However, none of the patients required blood patching, and headaches were treated with conservative treatment of postoperative hydration. Despite dural punctures, headaches were not seen in 3 patients. It may be that the fluid given to the epidural space during epiduroscopy prevented headache in these patients. Two of the patients who had complications developed foot drop. Symptoms in these patients were evaluated as transient neurological symptoms, and improvement was observed in subsequent follow-up for one of the patients. However, in the other patient, a foot drop finding continued as a permanent neurological deficit.

The dural puncture rates were changed between 0% and 21% during ELND, and the dural puncture rate was 7% in our study [13, 14, 19, 23].

Schütze [1, 2] reported that they were trying not to exceed 30 minutes of epiduroscopy in their clinical practice. In 2006, the consensus determined by the World Spine Endoscopy Initiative (WISE) Committee stated that the epiduroscopy procedure should not exceed 60 minutes [24]. The duration of epiduroscopy in our study ranged from 9 to 53 minutes with an average of 22.72 ± 9.35 minutes.

Unfortunately, none of the publications in the literature have compared the age of the patients undergoing epiduroscopy and the level of success of the procedure. In our study, results obtained by ROC analysis showed that the Holmium-YAG laser-assisted ELND method was more effective in patients under the age of 44 years.

Our study does have some limitations; however. it was conducted retrospectively and has the lack of a control group. But, this study was performed with a large patient population. Yet prospective, multidisciplinary studies are required.

As a result, ELND with Holmium: YAG laser, which is a new technique in patients with lumbar disc herniated low back and/or leg pain, can reduce VAS and ODI scores from 2 weeks without any complications that open surgery can bring. We believe that it is a useful and advanced technique in the treatment of lumbar disc herniation and has low complication rates that provide maximum efficacy from the first year. We think that prospective randomized controlled trials are needed to evaluate the efficacy, safety, complications, and long-term effects of this new technique of ELND and to compare it to other open surgical and minimally invasive techniques in lumbar disc herniation.

## Figures and Tables

**Table 1:** Distribution of demographic characteristics of patients.

Age (year)	48.62 ± 12.07
Age groups	
<35 years	19 (11.7)
35–44 years	47 (28.8)
45–54 years	48 (29.4)
≥55 years	49 (30.1)

Gender	
Female	92 (56.4)
Male	71 (43.6)

Weight (kg)	79.55 ± 13.99
Length (cm)	167.57 ± 10.30
BMI (kg/m^2^)	28.44 ± 5.13

Data are given as Minimum-Maximal (Median), Mean ± SD, and *n* (%). BMI: body mass index.

**Table 2:** Peroperative characteristics and complications of epiduroscopic discectomy procedures.

The amount of fluid (mL) given to the epidural space during epiduroscopy	133.42 ± 48.88
Epiduroscopy duration (min)	22.72 ± 9.35

Patients treated with lumbar epidural steroids	151 (92.6)

Patients treated with epiduroscopic laser	163 (100.0)

The amount of epiduroscopic laser (joule)	85–858 (273)
	312.31 ± 157.82

Complications	14 (80)

Dural puncture	12 (85)

Foot drop	2 (15)

Data are given as Minimum-Maximal (Median), Mean ± SD, and *n* (%).

**Table 3:** Evaluation of success and age relation.

	Preoperative-postoperative 12th month decrease (%)	Age (year)		*p*
		*n* = 163	Average ± SD	
VAS success	<%50	59	52.53 ± 10.30	0, 001^*∗∗*^
	≥%50	104	46.40 ± 12.48	

ODI success	<%40	43	53.46 ± 10.47	0.001^*∗∗*^
	≥%40	120	46.88 ± 12.17	

Student *t*-test. ^*∗∗*^ Statistically as significant, *p* < 0.001.

**Table 4:** Correlation between VAS and ODI success and age (cutoff value, 44).

		Age (year)				*p*
		<44 years		≥44 years	
		*n*	%	*n*	%
VAS success	≥%50	51	82, 3	53	52, 5	0, 001^*∗∗*^
	<%50	11	17, 7	48	47, 5	

ODI success	≥%40	55	88, 7	65	64, 4	0.001^*∗∗*^
	<%40	7	11, 3	36	35, 6	

Pearson chi-square test. ^*∗∗*^ Statistically as significant, *p* < 0.001. The ODDS for age at VAS success is 4,199 (95% CI: 1.964–8.975). For ODI success, the ODDS for age is 4.352 (95% CI: 1.795–10.551) (ODSS: relative/predicted risk ratio, CI: confidence interval).

## Data Availability

The [DATA TYPE] data used to support the findings of this study are available from the corresponding author upon request.
